# Assessing Alien Plant Invasions in Urban Environments: A Case Study of Tshwane University of Technology and Implications for Biodiversity Conservation

**DOI:** 10.3390/plants13060872

**Published:** 2024-03-18

**Authors:** Takalani Nelufule, Tinyiko C. Shivambu, Ndivhuwo Shivambu, Moleseng C. Moshobane, Nimmi Seoraj-Pillai, Tshifhiwa Nangammbi

**Affiliations:** 1 Department of Nature Conservation, Tshwane University of Technology, Private Bag X680, Pretoria West 0001, South Africa; 2 South African National Biodiversity Institute, Pretoria National Botanical Garden, 2 Cussonia Avenue, Brummeria, Silverton 0184, South Africa; m.moshobane@sanbi.org.za

**Keywords:** alien invasive plants, biological invasions, pathways, escapes, urban invasion dynamics, impact assessment, NEM:BA Regulations

## Abstract

Preserving the dwindling native biodiversity in urban settings poses escalating challenges due to the confinement of remaining natural areas to isolated and diminutive patches. Remarkably scarce research has scrutinised the involvement of institutions, particularly universities, in introducing alien plant species in South Africa, thus creating a significant gap in effective monitoring and management. In this study, the Tshwane University of Technology in Tshwane Metropole, South Africa serves as a focal point, where we conducted a comprehensive survey of alien plants both within the university premises and beyond its confines. The investigation involved the classification of invasion status and a meticulous assessment of donor and recipient dynamics. Our findings encompass 876 occurrence records, revealing the presence of 94 alien plant species spanning 44 distinct families. Noteworthy occurrences among the dominant plant families are Asteraceae and Solanaceae. Herbaceous and woody plants emerged as the most prevalent alien species, with common representation across both sampling sites. A substantial majority of recorded species were initially introduced for horticultural purposes (51%) before escaping and establishing self-sustaining populations (62%). Furthermore, 43 species identified are listed in South African invasive species legislation, with some manifesting invasive tendencies and altering the distribution of native species in the remaining natural areas. The notable overlap in species observed between the university premises and adjacent areas provides crucial insights into the influence of institutions on the dynamics of plant invasions within the urban landscape. This underscores the prevailing gaps in the management of invasive alien plants in urban zones and accentuates the imperative of an integrated approach involving collaboration between municipalities and diverse institutions for effective invasive species management in urban environments.

## 1. Introduction

Maintaining biological diversity in urban habitats has become a conservation priority while protecting the remaining natural areas within cities is viewed as more important than ever before [1,2,3,4]. However, conserving native biodiversity in urban areas faces a growing challenge as the remaining natural areas are often limited to isolated and small patches. These urban environments play an important role in society by providing benefits that sustain and improve the quality of life and human livelihood through urban ecosystem services (UESs) [5].

Urban habitats often accommodate fragmented native plant populations and face pressures from habitat destruction and small population sizes due to infrastructure development [6,7]. Additionally, urban areas face challenges like the increasing introduction of alien plants, migration, and resource-intensive commerce [8,9]. This trend of urban expansion and the transformation of natural landscapes poses significant threats to native species, leading to biodiversity loss and ecosystem disruption, making cities the global hotspots for the introduction of alien species [10,11].

Horticulture has been identified as a primary pathway for the introduction of alien plant species into urban areas, where they often escape from gardens and become invasive in natural habitats [9,12,13]. The extensive cultivation and ongoing introduction of numerous alien species for ornamental horticulture have resulted in a significant abundance of alien plant propagules [14]. This trend indicates that many species introduced in the past have the potential to become invasive over time, leading to a substantial invasion debt [15]. Additionally, these introductions are predicted to facilitate the expansion of invasive plant species in the future due to climate change [16]. Hence, it is important to monitor the distribution of alien plants in urban areas to identify those that are naturalising or becoming invasive.

Alien plants are often introduced to urban areas to offer urban ecosystem services (shade, aesthetics, and soil stabilisation) [17,18,19] that native species cannot provide as they often take longer to grow and are not as beautiful as plants introduced from other countries (i.e., *Jacaranda mimosifilia*). However, if not managed properly, alien plants can cause ecosystem disservices (EDSs) [20] such as heightened fire hazard [21], health concerns (i.e., allergenic pollen), safety hazards from falling trees, damage to infrastructure and properties, and reduced water flow, leading to biodiversity loss and risks to human well-being in the urban area [22,23,24,25,26,27]. These negative impacts have financial implications, for example, the costs of repairing damage to urban infrastructure and pruning plants [20,28]. Therefore, there is a need to identify potential threats posed by alien plants on the infrastructure and remnant natural habitat in urban areas.

As per the United Nations Environmental Programme [29], Aichi Biodiversity Target 9 emphasises the urgent need to document invasive alien species, reduce their populations, and eradicate them where possible [30]. Monitoring, rapid response, and early detection coupled with surveillance have proven effective in identifying alien and invasive species [31,32]. The implementation of these measures is crucial for compliance and ensuring that invasive alien species are eradicated before establishment, thus reducing the overall cost of management. South Africa’s National Environmental Management: Biodiversity Act (NEM:BA, Act 10 of 2004) mandates landowners including municipalities to control invasive species under their jurisdiction [33,34]. However, many municipalities lack the necessary human capacity, like invasion biologists, to effectively comply with the NEM:BA Regulations. To address this challenge, municipalities need to identify and monitor alien plants and their pathways of introduction [35].

In South Africa, the widespread distribution of invasive alien plant species poses a significant and escalating threat to urban biodiversity [36]. Urban ecosystems across the country, particularly in regions like Tshwane, are experiencing heightened levels of invasion, with these invasive plants predicted to continue expanding over the next three decades [37]. Despite being recognised as hotspots for biological invasions, urban areas in South Africa have received little attention directed towards understanding the pathways through which alien plants are introduced and the risks they pose to both urban and adjacent natural habitats. Existing studies have mainly focused on the dynamics of invasion, species impacts, and the associated challenges in protected areas, with limited attention to the specific pathways facilitating alien plant introductions into urban environments. Furthermore, research efforts have been concentrated in small towns and cities, with a notable scarcity of comprehensive studies in major urban centres like Tshwane [26]. This is concerning given the presence of Critical Biodiversity Areas (CBA 1) within Tshwane and six nature reserves, which conserve one of the largest peatlands in South Africa (https://www.tshwane.gov.za/, accessed on 7 December 2023), indicating a pressing need for focused investigation into the invasion dynamics within this urban context [38,39].

Despite the growing recognition of the invasive alien plant problem in South Africa, there is a significant knowledge gap regarding the specific pathways by which alien plants enter urban environments and the subsequent risks they pose to native biodiversity. To address these gaps, we used Tshwane University of Technology (TUT) as a case study, with the aims to (i) identify alien plant species and establish baseline data on alien plant species richness in terms of their families, invasion status, life forms, the continent of origin, and national invasive species legislation lists, (ii) point to primary pathways facilitating the introduction of invasive alien plant species into urban areas, (iii) assess the likelihood of the university to act as a donor to natural areas outside the campus (i.e., likelihood of escape), and (iv) make policy and management recommendations for the management of invasive species in the urban environment.

Due to the lack of an inventory of alien plants planted for horticulture during the time of this study, we predicted that alien plants would be distributed within the campus as they are likely to be treated as those that are used for landscaping purposes. We also predicted that the number of alien plants would be high outside the university compared to those found within the university because the latter are likely to be removed during garden services or maintenance. See the glossary below for a definition of the terms used in this paper (Table 1).

## 2. Results

### 2.1. Identification of Alien Plant Species and Families

**Figure 1:**
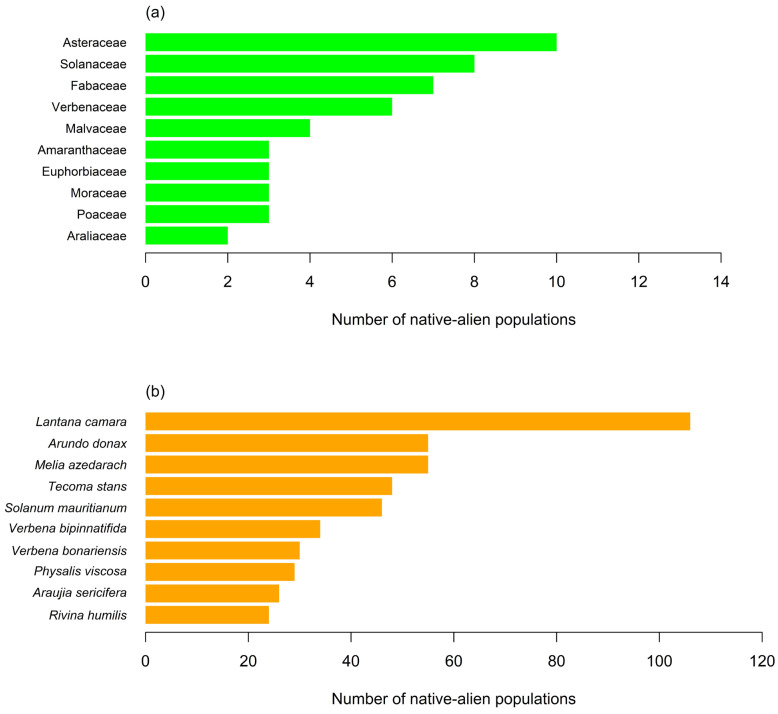
The top 10 most frequently recorded alien plant species at the Tshwane University of Technology and surrounding areas (**a**) ranked by the most dominant families and (**b**) the number of alien populations of the most abundant species.

We gathered 876 occurrence records encompassing 94 alien plant species distributed among 73 genera and 44 distinct families (Table A1). The richest family was Asteraceae, followed by Solanaceae, Fabaceae, and Verbenaceae (Figure 1a). No alien species was sharply prevalent over the others except for *Lantana camara* (Figure 1b).

### 2.2. Comparing Invasion Status, Life Forms, Continent of Origin, and NEM:BA A&IS Regulations Category

**Figure 2:**
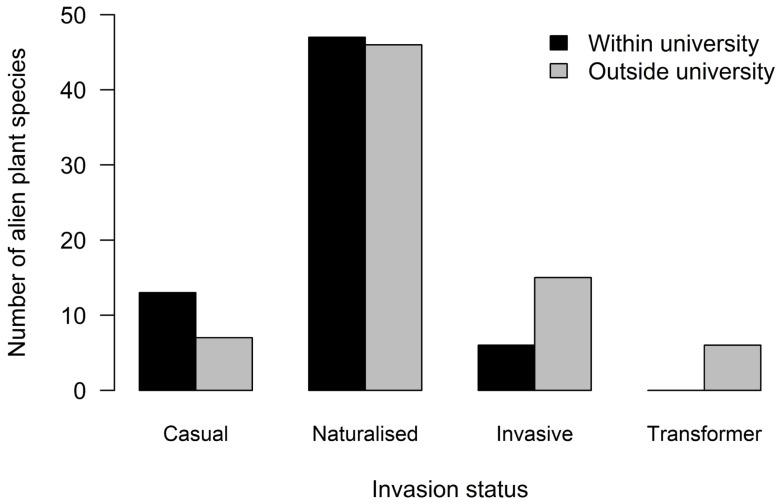
The number of alien plant species per invasion status within and outside the university campus.

In terms of invasion status, 15% of TUT species were casual aliens, 61% were naturalised aliens, 18% were invasive, and 5% were transformers from both within the university and natural areas outside the university (Figure 2). The number of species that had formed naturalised populations was significantly higher than those that had formed invasive, transformer, and casual populations (χ2 = 14.79, df = 1, *p* = 0.001; Supplementary Table S1a).

**Figure 3:**
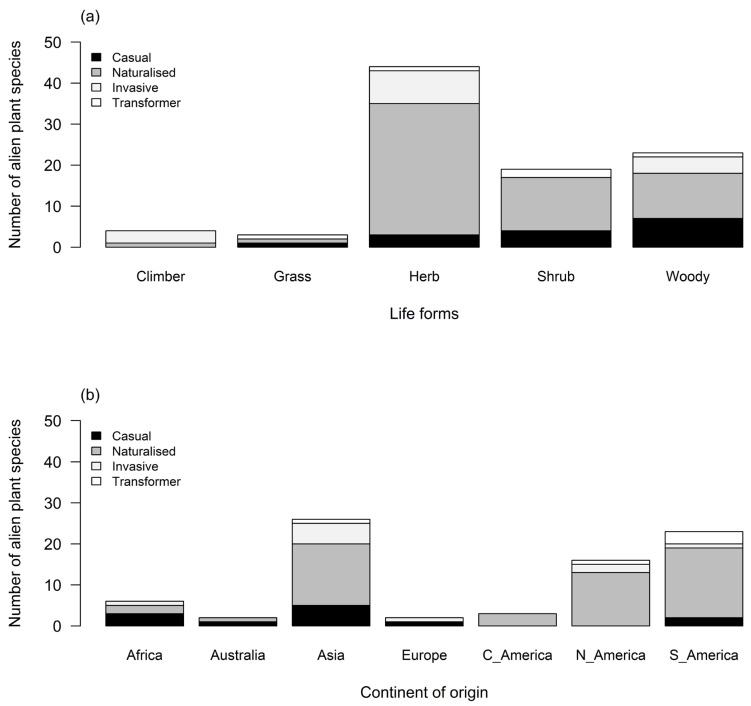
Stacked plot showing the number of recorded alien plant species present on the TUT campus categorised by (**a**) life forms and invasion status and (**b**) continent of origin and invasion status.

Herbs (47%) and woody alien plants (24%) were the most represented life forms, followed by shrubs (20%) (Figure 3a). We observed a significant difference between the number of herbaceous and woody species (χ2 = 4.28, df = 1, *p* = 0.038) and between the number of herbs and other life forms (*p* < 0.05; Supplementary Table S1b). The largest number of species originated from Asia (28%) and South America (24%), but few (*n* = 3) originated from Australia (Figure 3b). The number of species from Asia was significantly more numerous than species introduced from Africa, Central America, Europe, and Australia (*p* = 0.05; Supplementary Table S1c). However, there was no significant difference between the number of species introduced from Asia and South America (χ2 = 0.05, df = 1, *p* = 0.825) and between Asia and North America (χ2 = 1.50, df = 1, *p* = 0.221; Supplementary Table S1c).

**Figure 4:**
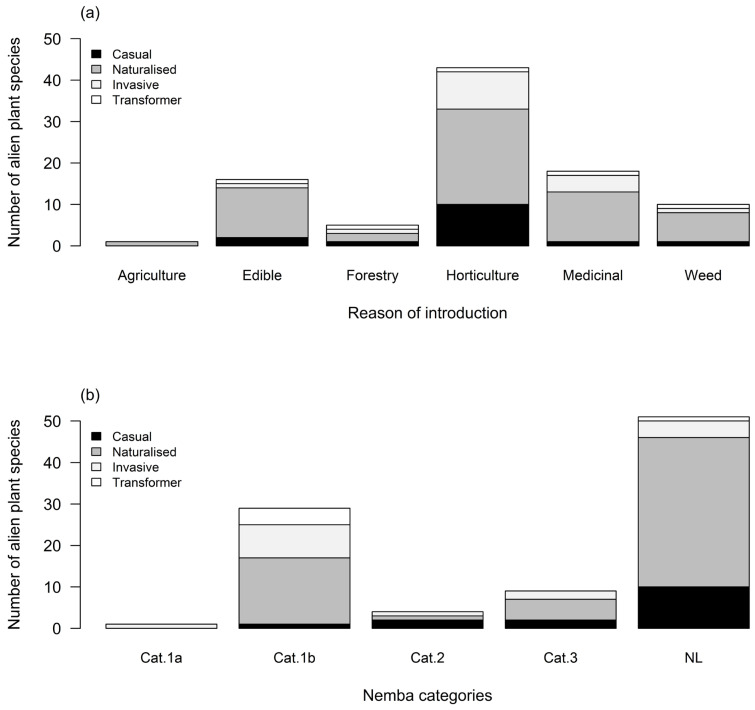
Stacked plot showing the number of recorded alien plant species present on the TUT campus categorised by (**a**) reason of introduction and invasion status and (**b**) the South African invasive species regulation categories and invasion status.

A large number of species were introduced for horticulture (*n* = 43) than for the other pathways, with the majority forming naturalised populations when they were introduced (Figure 4a). The number of species that were introduced for horticulture was significantly higher than the number of species introduced for medicinal purposes (χ2 = 7.009, df = 1, *p* = 0.008), edible (χ2 = 9.519, df = 1, *p* = 0.002) and for forestry, agriculture, and weeds (*p* = 0.001) (Supplementary Table S1d).

A total of 43 out of 94 alien plant species are listed in the South African invasive species legislation regulations list (NEM:BA A&IS Regulations)—31% being Category 1b, 3% Category 2, and 9.5% Category 3 invaders (Figure 4b). Many species that had formed naturalised populations were not listed under the NEM:BA regulation categories, followed by those classified as Category 1b (Figure 4b). Finally, the number of species that were not listed was significantly higher than the number of species listed under Categories 1a, 1b, 2, and 3 (*p* < 0.05; Supplementary Table S1e). Only one species, *Pueraria monatana*, was listed in Category 1a (Figure 4b).

### 2.3. Species Distribution between Sampling Sites

**Figure 5:**
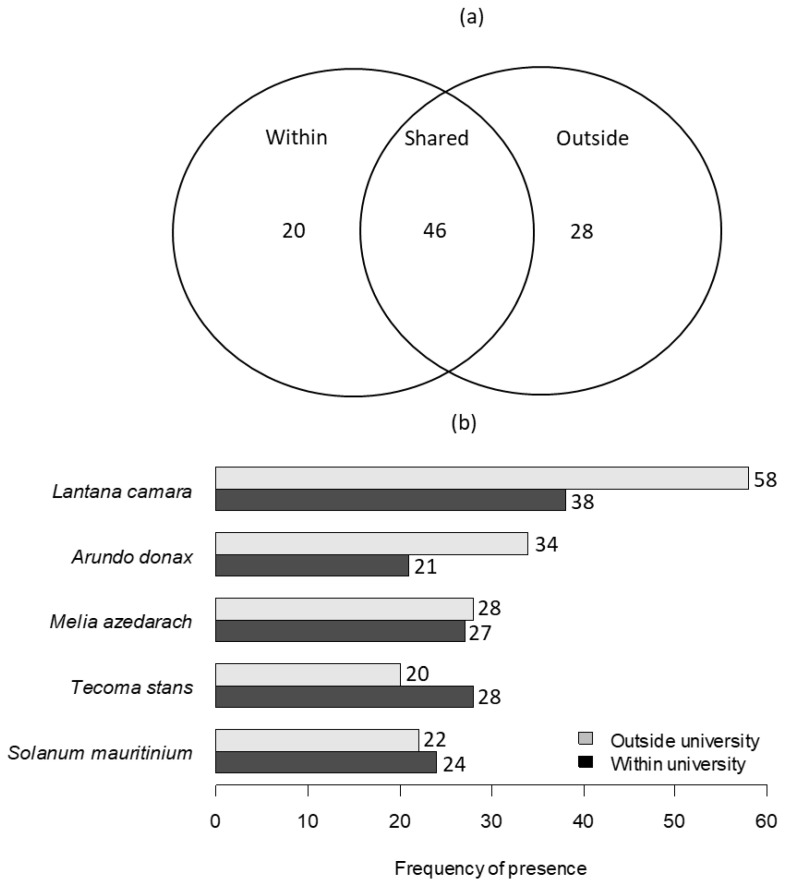
A Venn diagram showing (**a**) the number of alien species detected only within the TUT campus (**left**), only outside the fence (**right**), and at both sites (**centre**); (**b**) frequency of presence for the top five species shared between the university and areas outside the university.

The number of species recorded within the university was not significantly different from those recorded outside the university fence (χ2 = 0.1603, df = 1, *p* = 0.6889). However, the abundance of species recorded outside the university fence was significantly higher (*n* = 485) than those recorded within the university campus (*n* = 391) (χ2 = 6.52, df = 1, *p* = 0.010). A total of 20 species (21%) were recorded within the university only, 30% outside of the university, and 49% were shared between the two sampling sites (Figure 5a). The top five most frequently recorded species were the same between the two sampling sites but varied in the number of times each species was recorded, namely, *Lantana camara*, *Arundo donax*, *Melia azedarach*, *Tecoma stans*, and *Solanum mauritianum*, respectively (Figure 5b).

## 3. Discussion

### 3.1. Species Composition

**Figure 6:**
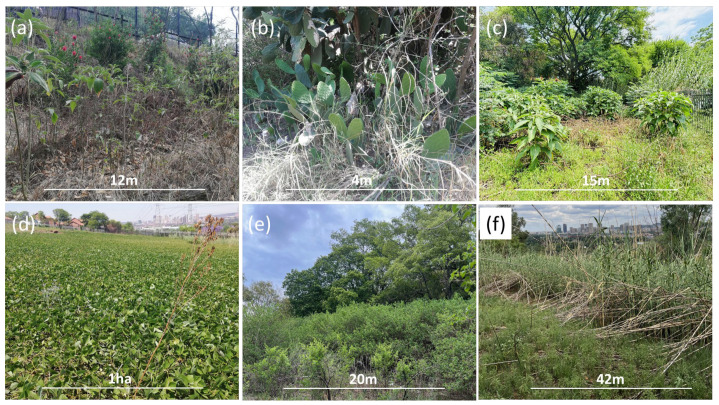
(**a**) Populations of *Solanum mauritianum* and *Callistemon viminalis* outside the university fence; (**b**) population of *Opuntia ficus-indica* in the natural area outside the university; (**c**) population of *Arundo donax* with *Solanum mauritianum* and *Melia azedarach* within the university campus; (**d**) a spreading population of *Pueraria montana*; (**e**) invasive populations of *Lantana camara*; and (**f**) *Arundo donax* replacing native species outside the university (photo cred: Takalani Nelufule).

The Asteraceae, Solanaceae, Fabaceae, and Verbenaceae families dominated the alien plant pool. The dominance of Asteraceae suggests the need for management strategies that target species within this family, while the prominence of the genera *Verbena* and *Solanum* indicates a potential impact on the local biodiversity and the adaptability of these groups. In addition, Asteraceae has been shown to be a dominant plant family in other studies [42], and their dominance in natural areas in our study area is of no surprise [43]. Alien plant species reported here represent 10% of the overall alien plants found in South Africa and 10% (*n* = 25) of invasive species recorded in Gauteng Province [44]. About 30% of these species have not been recorded for Tshwane regions in the updated version of the Southern African Plant Invaders Atlas (SAPIA) database [45]. This highlights the critical role of monitoring alien plant species in urban areas, as demonstrated by this study. The present study identifies alien plant species that should be included in the SAPIA records for the Tshwane region, thereby contributing to the national database of invasive alien species as urban areas are identified as key points for the introduction of alien plants. The number of species recorded in the natural areas outside the university was high compared to the alien plants (*n* = 43) recorded at the urban/wildland interface on the Cape Peninsula, South Africa [46]. High plant taxonomic richness relative to the area sampled (Figure 6) indicates the diversity of alien plants in Tshwane and shows that the remaining natural areas in the urban areas are vulnerable to invasion. The widespread distribution of *Pueraria montana*, *L*. *camara*, and *A*. *donax* highlights the gap between the local authorities (local municipalities and government institutions) and institutions in managing invasive alien species in the urban area (Figure 6d–f).

Seven newly reported native-alien populations recorded in this study will add to the list of native-alien populations in South Africa reported by Nelufule et al. [47]. Two of these species were shared between the two sampling sites, namely, *Aptenia cordifolia* and *Tephrosia grandiflora*, while five other species were recorded only within the university campus. This indicates an increase in the movement of native species outside their historic native range and the lack of reporting of this phenomenon globally [48]. We encourage the landscaping services at different institutions to use native plant species from the same biogeographical region for ornamental or landscaping purposes, as native species pose risks of forming native-alien populations in different countries and have been reported to threaten biodiversity [49,50].

### 3.2. Invasion Status

Many alien plant species documented both within the university and outside had established naturalised populations and showed invasive behaviour in natural areas outside the university. This included five species identified as “transformers” (see Figure 6), all observed thriving in natural areas outside the university premises. Invasive populations include damaging alien woody plant species such as *L*. *camara*, which is one of the worst alien invasive species in the world [51]. This indicates the undocumented impacts of alien plants upon native vegetation abundance and species composition in natural areas through competition as this species is replacing natural vegetation and raises concerns for other natural areas in urban environments [52]. A plausible explanation of this widespread presence of alien plants outside the university fence could be due to the limited capacity to implement control measures of biological invasions at the university [53], long residence time [54], unmanaged pathways associated with human activities (e.g., illegal dumping and horticultural practices), high propagule pressure from the university, and disturbed soil as a result of fence construction, which favours the establishment of alien species [54,55,56,57].

Species that have formed casual populations have not reached their full invasion potential due to invasion debt [15], but this could change if the propagule pressure increases [58]. A large number of naturalised alien plant populations within the university could be explained by the huge portion of disturbed soil created during the construction of the university and the availability of water, which is used to water garden plants. This also means that these species were introduced within the university a long time ago and overcame a series of biotic and abiotic barriers that prevented their survival. Therefore, institutions are encouraged to manage their gardens and soils so that it does not lead to the establishment of alien plants.

**Figure 7:**
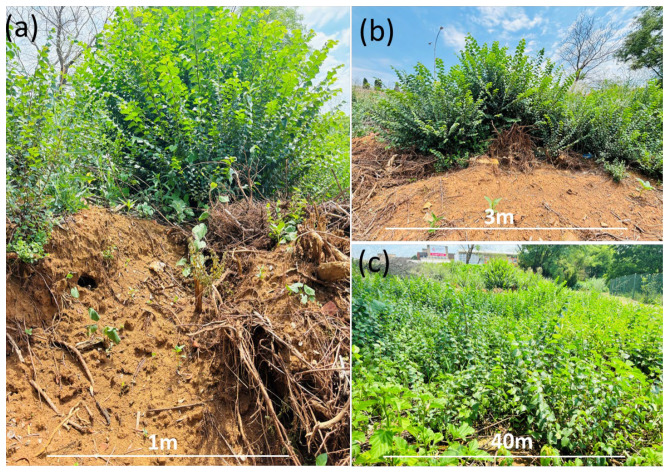
Picture showing (**a**,**b**) an attempted eradication of (**c**) the spreading population of *Ulmus pumila* within the university campus (photo cred: Takalani Nelufule).

There is an urgent need for institutions to educate horticultural or landscaping operators about the common alien and invasive species so that the abundance and spread of invasive alien plants can be reduced in urban landscapes. This will help reduce the establishment of unwanted alien species and their potential escape into other urban environments. The establishment and spread of *Ulmus pumila* within the university highlight the role of institutions in introducing alien plant species and providing a suitable habitat for them to spread (Figure 7). Institutions should, therefore, conduct a thorough risk assessment for each species before introducing them for landscaping or horticultural purposes. A spreading population of *Ulmus pumila* was also recorded outside the university fence, which suggests that institutions act as a source of alien plant introduction in urban areas. The widespread distribution of invasive species like *L*. *camara*, *U*. *pumila*, and *A*. *donax* highlights the gap in the management of invasive species in the urban habitat and requires urgent collaboration between institutions and authorities working on the management of invasive species (i.e., local municipalities and government agencies).

### 3.3. Life Forms

Herbaceous growth forms accounted for the majority of species that had formed alien plant populations in this study. Of the recorded herbaceous species, 72% were naturalised populations and 18% were invasive (Figure 4a). Forty-seven percent of woody species had formed naturalised populations, while 21% formed invasive populations. The dominance of herbs and woody plants indicates that plants from these two life forms have successfully adapted to their new environment. Therefore, herbaceous alien plant species are more likely to become successful invaders in natural areas and spread rapidly because of their shorter generation times [59]. This underscores that herbaceous species are great invaders of urban areas [36]. Our findings could be attributed to various factors such as favourable environmental conditions, a vast portion of disturbed habitat in the urban area favouring the establishment of herbaceous plants, the common use of ornamental herbaceous species for horticultural services within the university, and accidental introduction with nursery soil [60,61]. The proportionally large number of woody species that had formed invasive species corresponds to the prevailing notion that woody species or trees are dominant invaders [62,63]. Most of the woody alien plants that become invasive have caused massive impacts in their introduced range [21,64,65]. This indicates that woody species present threats to the university’s infrastructure, the natural areas outside the university, and nearby suburbs [66]. This could imply that the university is compelled to allocate substantial unbudgeted funds towards removing alien woody plants and subsequent repair of infrastructure damaged by these species. For example, we observed a spreading population of *Ulmus pumila* that prompted the university to remove the population, although the eradication does not appear to have been successful (Figure 7). This study shows the most adapted alien plants in urban environments. Herbaceous alien plants should, therefore, be managed in a way that minimises their escape from gardens in areas where they are used for landscaping purposes in the urban environment.

### 3.4. Continent of Origin

The majority of naturalised alien plants originated from Asia and South America but very few species were introduced from Australia, indicating the global movement of plants. Most species that had formed invasive populations originated from Asia, while the transformer species originated from South America. The large number of species introduced from Asia could be explained by the import of live plants from China to South Africa [67]. In contrast, the low number of species from Australia could be attributed to their effective biosecurity measures [68]. The large number of alien plants introduced from North America could be linked to the popularity of ornamental plants and the large number of nurseries selling invasive alien species on the Internet, which could have allowed some of these species to reach South Africa [16]. Therefore, there is a need to increase surveillance in the horticulture trade in South Africa so that potential invasive species can be identified before they can be introduced into urban areas and then into the wild [69]. Although only five species had been introduced from other African countries, we expected these numbers to be high because there has been an increase in trade between South Africa and other African countries [70], meaning that it is likely that there has been an increase in the number of alien taxa from these countries.

### 3.5. National Listing (NEM:BA A&IS Category)

Different listed species, including unlisted species (e.g., Categories 1b, 2, and 3) indicate the varied degrees of threat that require conservation strategies. Similarly, other studies have reported that the majority of alien plants found in urban areas [46] were not listed in the South African invasive species regulation lists. This could be due to the limited capacity to perform alien taxa risk assessments at a regional level and by local municipalities [35,71]. Species that are not listed, especially those that are naturalised and show signs of invasiveness (e.g., *Ulmus pumila*), require further assessment using the standardised impact assessment framework [71] so that their impacts can be known and the species avoided for use by horticultural and landscaping services.

One species was identified as an emerging invader targeted for early detection and rapid response (Category 1a) in South Africa [32], however, the species was not under any form of management at the time of the survey. Emerging invader plants have also been recorded in other urban areas in Cape Town and Nelspruit in South Africa [72], which indicates that urban areas provide suitable environments for alien plants to complete invasion continuum. This is because many alien plants are first introduced in urban areas and then move into the wild [73]. Our study highlights the significance of conducting surveys in urban areas as they aid in identifying species in need of urgent management attention and contribute to updating the inventories of invasive species at both the local and national levels. There is a need for continuous assessment of alien species in urban areas globally so that threats to native biodiversity and the socio-economy can be identified before species become widely invasive.

### 3.6. Species Shared between Sampling Sites

Despite the garden maintenance efforts within the university, many of the species found within the university had formed naturalised populations, suggesting a prolonged residence time and inadequate gardening services due to a lack of awareness and knowledge regarding alien plant species. For example, patches of alien plants were left standing within the university during garden maintenance, which allowed these species to spread further (see Figure 6). The abundance of alien plant species outside the university was attributed to the lack of garden maintenance outside the university fence. Our results emphasise that half of the species recorded are shared between the university and the surrounding areas and indicate a widespread presence and potential spread or spill-over between the two sites. The top five most frequently recorded species were the same between the two sampling sites. The existence of shared species within the university and the natural areas outside the university fence underscores the urgent need to control the spread of these common invasive plants. We propose that institutions, including the TUT, compile a list of alien plant species that are used for landscaping and update their list so that species that were not planted can be removed. This will prevent the escape of alien plants into the natural areas and urban spaces outside the university.

### 3.7. Reason of Introduction

This study shows that, among the species introduced for horticultural purposes, 41% had established naturalised populations, while 18% were classified as invasive (Figure 4a). Our results emphasise the potential ecological risks linked to horticultural introduction and could also mean that the alien plants recorded here likely arrived within the university for landscaping or horticultural purposes, then escaped from cultivation or gardens. This study shows the increasing role of horticulture in facilitating plant introductions and invasions [60,74]. Other studies in South Africa have linked the introduction of ornamental plants with the horticultural trade in small towns in Western Cape Province and elsewhere worldwide [75,76]. The proportion of naturalised medicinal and edible plants reflects the variety of active pathways that are introducing alien plants and the involvement of humans in introducing alien species in urban areas. In addition, some of these plant species could have been introduced in the study area naturally by birds (e.g., fleshy-fruited species) [77], through railway lines passing near the university fence and connecting the university with other areas within the city of Tshwane (e.g., gardens) [21,53], and runoff water from areas outside the university, which suggest re-invasion. Naturalised weeds are most likely to have been introduced as containments as these species have been moved around the world for many years, for example, *Solanum elaeagnifolium* [78].

Most South African universities are situated in urban areas, making them an ideal source of alien plant propagule pressure [58]. Universities, therefore, provide a launching site for alien plant invasion in urban areas due to the common practice of using ornamental alien landscaping within their campuses. To prevent the further spread of alien plant species in urban areas, institutions must ensure that their horticulture service providers comply with national invasive alien species regulations and other laws that govern them (i.e., the code of conduct on horticulture and invasive alien plants). This can be achieved through education, monitoring, and enforcement.

### 3.8. Recommendations

We observed the common use of fire within and outside the university during the survey, with this practice likely creating a suitable habitat for the establishment of species like *Populus alba*, *Arundo donax*, *Physialis viscosa*, and *Ulmus pumila*, which have a widespread distribution outside the university in natural areas and are replacing native vegetation. This could be because native grass species take a long time to recover after fire [79,80]. Wildfire has been reported to promote the dominance of invasive species such as the giant reed (*Arundo donax*), for example, in California, North America [81], which explains the widespread distribution of *A*. *donax* within and outside the university in the natural areas including other parts of the city of Tshwane. Therefore, we propose that institutions, through their landscaping or garden services, review their practices of using fire as a management tool and use a species-based management approach when managing alien invasive plants. The lack of appropriate management of these invasive alien plants in natural areas will increase the expansion of alien plant infestations and reduce local biodiversity [82,83].

### 3.9. Management Implications

The Early Detection and Rapid Response (EDRR) constitutes a pivotal facet in the overarching framework for the management of alien and invasive species in South Africa. However, the ubiquity of this surveillance imperative demands a considerable allocation of resources, particularly in the context of developing nations, wherein the enforcement of such a strategy can prove inherently burdensome. Consequently, it is propounded that the Biodiversity Management Authority within the Tshwane Metropolitan Area undertakes the establishment of localised task groups or networks. These networks are envisioned to serve as vigilant entities, tasked with the systematic monitoring and expeditious reporting on the management of invasive alien species (IAS) within distinct local municipalities. Comprising a heterogeneous assemblage of stakeholders including individual organisations, corporate entities, private sector entities, government sectors, professionals, and environmental enthusiasts, these localised networks converge upon a shared objective, namely, the early detection of alien species within their respective domains. This collaborative initiative thereby operates in consonance with the imperative to alleviate the resource-intensive challenges associated with nationwide surveillance.

Drawing a parallel from the French working group on biological invasions in freshwater environments, it becomes evident that such initiatives crystallise around a shared interest in mitigating the deleterious impacts wrought by IAS within the confines of a given province [84,85]. The augmentation of surveillance capacity, heralded by an increased number of vigilant entities, serves as a bulwark against the proliferation of extant and emerging invasive alien species infestations. This heightened state of vigilance predicates the expeditious reporting of identified incursions to the pertinent authorities, catalysing prompt and efficacious responsive actions [84,85,86,87].

## 4. Materials and Methods

### 4.1. Geographical Study Site

**Figure 8:**
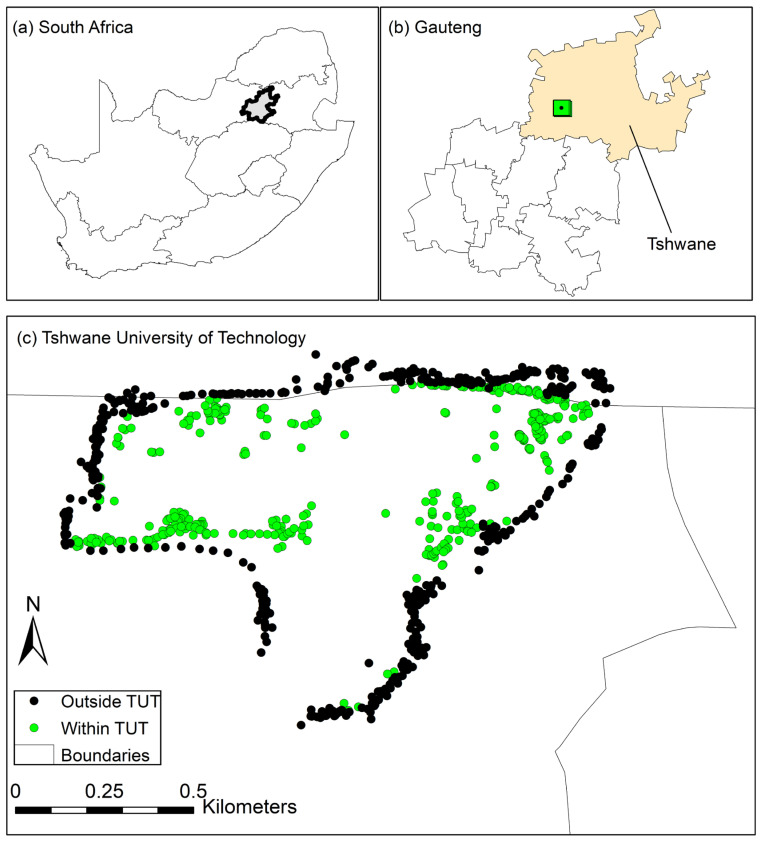
Map showing South Africa and Gauteng Province in grey colour (**a**), Gauteng Province and Tshwane municipality including study site in green colour (**b**), and Tshwane University of Technology with sampling sites within the university in green and outside the university in black dots (**c**).

Our study was based at the Tshwane University of Technology (25.7322° S 28.1619° E), located on the lower slopes of Witwatersberg in the urban area of Tshwane in Gauteng Province, South Africa (Figure 8). The dominant natural vegetation types in the area are Savanna [88], which is classified as Clay Thorn Bushveld, and Moist Clay Highveld Grassland [89], both present at a distance of few kilometres from the Pretoria National Zoological Gardens. The altitude of the study area is between 1400 and 1800 m above sea level. The main geological formations are the Daspoort, Timeball Hill, and Magaliesberg Formations from the Pretoria Group.

### 4.2. Data Collection and Species Selection

To survey alien plants within the university, a map of the university was downloaded from the Internet and sampling blocks demarcated. Each building block, road, and area along the fence was surveyed for the presence of alien plants that were not deliberately cultivated for landscaping within the university campus. All ornamental alien plants that had been planted and cared for were excluded from this survey. These are plants cultivated by the horticultural or landscaping services at the university. We also surveyed areas outside the university fence following a belt transect (i.e., from 2 up to 50 m, where access was not possible due to dense stands of plants). This distance was chosen because alien plants can be dispersed at a greater distance from a propagule source by wind or water [90]. Four kilometres of transect outside the university fence were surveyed for alien plants. Both surveys were conducted over a period of seven days in November 2023, with three days spent within the university campus and four days outside the university, each day requiring a minimum of three hours of data collection. A follow-up survey was conducted in January 2024 for one day to confirm the identification of species and the status of some populations. We recorded the presence of all alien plant species visible within the transect outside the university fence, taking a GPS waypoint every 10 m [75,91]. We also recorded native-alien populations (i.e., South African native species occurring outside their geographical barriers, as indicated by Nelufule et al. [47]) and classified them using a protocol for identifying native-alien populations [92].

Following the criterion described in Chatterjee and Dewanji [36], each plant species was classified by life form as follows: climber, herbs, grass, shrubs, and woody. This information is useful as it can provide methods for predicting impact, although the habitat and invaded geographical region is not stated [93]. Furthermore, to understand and manage the pathway of introducing alien species, it is important to determine the region from where alien species are introduced [94]. Therefore, we also included the continental origin of each species as per the Centre for Agriculture and Bioscience International (CABI: https://www.cabi.org/, accessed on 30 November 2024) and the Global Invasive Species Database (GISD: https://www.iucngisd.org/gisd/, accessed on 30 November 2024) as we assumed that all species were introduced directly from their native country of origin into South Africa.

### 4.3. Species Verification and Identification

All specimens were identified in the field using field guidebooks [95,96] and photographs of each species were taken with geolocations for further identification. Species that could not be verified during the survey were pressed and sent to an expert at the South African National Biodiversity Institute’s National Herbarium (PRE) to be verified against specimens already logged. Species names were verified and assigned following the Plants of the World Online (POWO: http://www.plantsoftheworldonline.org/, accessed on 30 December 2023) and Global Biodiversity Information Facility Backbone Taxonomy (GBIF: https://www.gbif.org/dataset/d7dddbf4-2cf0-4f39-9b2a-bb099caae36c, accessed on 30 December 2023). In cases where specimens could not be identified to the species level because of a lack of taxonomic characteristic features such as fruits or flowers, such specimens were identified to the genus level. This information was included as it is important for estimating the richness of an alien species.

### 4.4. NEM:BA A&IS Category, Invasion Status, and Pathway Classification

Species were also categorised under the South African invasive species legislation (NEM:BA A&IS Regulations of 2021) (Table A1). The NEM:BA A&IS Regulations were promulgated in 2014, but we used the 2021 lists as they were more relevant during our survey. To avoid allocating one species into more than one category, the most common category for each species or area where a species is a priority for management was used as the correct category in this study [53]. We classified the invasion status of the recorded species as per Pyšek et al. [40] and Richardson et al. [97] and categorised species as casual alien, naturalised, invasive, or transformer.

Each species was also classified according to its likelihood of introduction, and this information was used as a proxy of the pathway of introduction following Baard et al. [49], where ‘A’ was for Agriculture; ‘E’ for Edible plant, ‘F’ for Forestry (including for the stabilisation of sand); ‘G’ for Grazing; ‘H’ for Horticulture; ‘M’ for plants known to have Medicinal use, and ‘W’ for Weed (where other modes are unlikely).

To determine the donor and recipient dynamics or possibility of the species escaping from the university, a Venn diagram was used to show the number of species shared within the university and areas outside the university fence. This was carried out by comparing the number of unique species on and off campus and those shared between the two sampling sites. Species richness was also used to calculate the number of species shared between the two sampling sites. We calculated the species richness or frequency of presence by counting the number of times each alien plant was recorded during each sampling point and used the top five most sampled species from both sampling sites to show this relationship.

### 4.5. Statistical Analyses

All statistical analyses performed in this study were constructed using the R statistical environment (version 4.2.2) [98]. Proportional statistics were calculated to understand the composition and structure of the alien plant diversity at the Tshwane University of Technology. To ascertain if the datasets utilised in this study were parametric, the Kolmogorov–Smirnov normality test was employed (i.e., independent, normally distributed, and identical). The datasets were, therefore, non-parametric (D = 0.977; *p* < 0.001) for the Kolmogorov–Smirnov normality test. To compare the number of alien species in different classification categories (i.e., life form, species continental origin, reason for plant species introduction (pathway)) and the categories of the NEM:BA A&IS Regulations, species count data were analysed using Chi-square tests (χ^2^), and Fisher’s exact tests were used in instances where the expected values were less than 4 (see Crawley) [99]. Alien plant species counts were generated from the total number of species obtained from the boundaries within and outside the university fence and specified as the dependent variable. We also tested whether there was a statistical difference between the number and abundance of species recorded within and outside the university fence using the Chi-square test. The statistical significance was accepted at *p* < 0.05.

## 5. Conclusions

Our study revealed the contribution of institutions in facilitating alien plant invasion in the natural areas within urban environments and offers a valuable understanding of the composition and invasion status of alien plants in one of the remaining natural areas within Tshwane. The richness of alien plant species was shared between various plant families. The distribution of invasion status differed amongst the invasion categories, suggesting the adaptability and likelihood of these species becoming invasive. Life forms showed the alien plant species that are mostly preferred for landscaping services by the institutions in urban environments. The majority of species that were recorded were introduced for horticulture and originated from South and North America and Asia, which highlights that alien plant invasion in this region is influenced by the global movement of plants through horticulture. There is a regulatory implication for some urban areas in South Africa, as 31% of species requiring total control were identified in the natural areas that were not being managed. The species shared between the sites indicate the widespread and possible interchange of alien plants between the university and natural areas within urban environments. This suggests that natural areas within urban areas are at risk of alien-plant invasions from horticultural and landscaping services. This study highlights the need for active measures to manage the pathways of alien species introduction and the necessity of reducing the impact of alien plant species on a region’s biological diversity and urban ecosystem. This study reveals the gaps between the management of invasive alien plants in urban areas and underscores the importance of an integrated approach to the management of invasive species between municipalities and various institutions in urban environments. We hope that this study will help increase the surveillance of alien plant species in the city of Tshwane.

## Figures and Tables

**Table 1:** Terms used in this study.

Casual	Alien plants that may flourish and even reproduce occasionally outside cultivation in an area, but eventually die out because they do not form self-replacing populations and rely on repeated introductions for their persistence [40].
Category 1a	Species that must be combatted or eradicated [33].
Category 1b	Species that must be controlled [33].
Category 2	Any species listed under Category 2 requires a permit issued by the Department of Forestry, Fisheries, and the Environment (DFFE) to carry out a restricted activity [41].
Category 3	Category 3 listed invasive species are subject to certain exemptions in the NEM:BA Act, which applies to the listing of alien invasive species [33].
Established	Alien plants that sustain self-replacing populations for at least 10 years without direct intervention by people (or in spite of human intervention) by recruitment from seed or ramets (tillers, tubers, bulbs, fragments, etc.) capable of independent growth [40].
Introduced range	Area outside the species’ historic native range where a species is introduced by human agency [40]
Invasion debt	“Invasion debt represents the additional number of future invasive species originating from the introduced species pool” [15].
Invasive	“Invasive plants are a subset of naturalised plants that produce reproductive offspring, often in very large numbers, at considerable distances from the parent plants, and thus have the potential to spread over a large area” [40].
Native-alien population	“A population that results from human-mediated dispersal of individuals of a taxon over a biogeographical boundary to a point beyond the taxon’s native range that is still within the same political entity as some parts of the taxon’s native range” [41].
Transformer	“A subset of invasive plants (not necessarily alien) that change the character, condition, form or nature of ecosystems over a substantial area (substantial means relative to the extent of that ecosystem)” [40].

## Data Availability

Data are available on request.

## Supplementary Materials

The following supporting information can be downloaded at: https://www.mdpi.com/article/10.3390/plants13060872/s1, Table S1. Output of Chi-square tests, comparing the number of species obtained from TUT across (a) invasion status category, (b) life forms, (c) continent of origin (d) reason of introduction and (e) categories from the NEM:BA A&IS Regulations.

## Appendix A

**Table A1:** List of alien and native-alien plant taxa recorded at the TUT with each species classified according to family, abundance, life form, categories from the NEM:BA A&IS Regulations, and invasion status defined by Pyšek et al. [40] and Richardson et al. [97]. Species with an asterisk are those that are shared within the university and outside the university fence. NL = not listed.

Species Name	Abundance	Family	Life Form	NEM:BA A&IS Categories	Invasion Status
*Albuca abyssinica* Jacq.	2	Hyacinthaceae	Succulent	Native-alien	Naturalised
*Abutilon grandifolium* (Willd.) Sweet *	7	Malvaceae	Shrub	NL	Naturalised
*Abutilon theophrasti* Medik.	3	Malvaceae	Herb	NL	Naturalised
*Agave americana* L.	1	Asparagaceae	Shrub	Category 3	Naturalised
*Ageratina adenophora* (Spreng.) R.M.King & H.Rob.	1	Asteraceae	Shrub	Category 1b	Naturalised
*Amaranthus* sp.	1	Amaranthaceae	Herb	NL	Naturalised
*Amaranthus spinosus* L.	2	Amaranthaceae	Herb	NL	Naturalised
*Anredera cordifolia* (Ten.) Steenis *	6	Basellaceae	Climber	Category 1b	Invasive
*Araujia sericifera* Brot *	26	Apocynaceae	Climber	Category 1b	Invasive
*Argemone mexicana* L. *	9	Papaveraceae	Herb	Category 1b	Invasive
*Arundo donax* L. *	55	Poaceae	Grass	Category 1b	Transformer
*Bidens bipinnata* L.	1	Asteraceae	Herb	NL	Naturalised
*Bidens pilosa* L. *	9	Asteraceae	Herb	NL	Invasive
*Callistemon viminalis* (Sol. ex Gaertn.) G.Don *	3	Myrtaceae	Woody	Category 3	Invasive
*Campuloclinium macrocephalum* (Less.) DC. *	6	Asteraceae	Herb	Category 1b	Invasive
*Canna indica* L. *	2	Cannaceae	Herb	Category 1b	Naturalised
*Celtis sinensis* Pers. *	4	Cannabaceae	Woody	NL	Naturalised
*Cenchrus setaceus* (Forssk.) Morrone *	4	Poaceae	Grass	NL	Naturalised
*Cestrum parqui* (Lam.) L’HÃ©r.	13	Solanaceae	Shrub	Category 1b	Naturalised
*Chenopodium album* L.	1	Amaranthaceae	Herb	NL	Naturalised
*Datura stramonium* L. *	5	Solanaceae	Herb	Category 1b	Naturalised
*Eucalyptus camaldulensis* Dehnh.	5	Myrtaceae	Woody	Category 1b	Naturalised
*Euphorbia heterophylla* L. *	4	Euphorbiaceae	Herb	NL	Naturalised
*Euphorbia* sp.	1	Euphorbiaceae	Herb	NL	Naturalised
*Ficus carica* L.	3	Moraceae	Woody	NL	Naturalised
*Flaveria bidentis* (L.) Kuntze	1	Asteraceae	Herb	Category 1b	Naturalised
*Galinsoga parviflora* Cav.	1	Asteraceae	Herb	NL	Naturalised
*Grevillea robusta* A.Cunn. ex R.Br.	1	Proteaceae	Woody	Category 3	Casual
*Hedera helix* L.	1	Araliaceae	Climber	Category 3	Naturalised
*Heliotropium amplexicaule* Vahl *	16	Boraginaceae	Herb	NL	Invasive
*Hirschfeldia incana* (L.) Lagr.-Foss.	1	Brassicaceae	Herb	NL	Naturalised
*Indigofera tinctoria* L.	1	Fabaceae	Shrub	NL	Casual
*Ipomoea purpurea* (L.) Roth	4	Convolvulaceae	Climber	Category 1b	Naturalised
*Iris ensata* Thunb *	1	Iridaceae	Herb	NL	Casual
*Iris* sp.	1	Iridaceae	Herb	NL	Casual
*Jacaranda mimosifolia* D.Don *	20	Bignoniaceae	Woody	NL	Naturalised
*Kraussia floribunda* Harv.	1	Rubiaceae	Shrub	Native-alien	Casual
*Lactuca serriola* L. *	13	Asteraceae	Herb	NL	Invasive
*Lantana camara* L. *	96	Verbenaceae	Shrub	Category 1b	Transformer
*Lantana trifolia* L.	4	Verbenaceae	Shrub	NL	Naturalised
*Ligustrum japonicum* Thunb *	7	Oleaceae	Shrub	Category 1b	Naturalised
*Ligustrum ovalifolium* Hassk.	2	Oleaceae	Shrub	Category 1b	Naturalised
*Malva parviflora* L.	1	Malvaceae	Herb	NL	Naturalised
*Malvastrum coromandelianum* (L.) Garcke	7	Malvaceae	Herb	Category 1b	Invasive
*Medicago lupulina* L.	1	Fabaceae	Herb	NL	Naturalised
*Melia azedarach* L. *	55	Meliaceae	Woody	Category 3	Invasive
*Melilotus albus* Medik *	8	Fabaceae	Herb	NL	Naturalised
*Aptenia cordifolia (L.f.) Schwantes*	2	Aizoaceae	Succulent	Native-alien	Naturalised
*Mirabilis jalapa* L. *	14	Nyctaginaceae	Herb	Category 1b	Naturalised
*Morus alba* L. *	20	Moraceae	Woody	Category 3	Invasive
*Morus rubra* L. *	16	Moraceae	Woody	NL	Naturalised
*Nandina domestica* Thunb.	1	Berberidaceae	Shrub	NL	Casual
*Oenothera rosea* L’HÃ©r. ex Aiton *	20	Onagraceae	Herb	NL	Naturalised
*Oenothera tetraptera* Cav.	4	Onagraceae	Herb	NL	Naturalised
*Opuntia ficus-indica* (L.) Mill. *	4	Cactaceae	Shrub	Category 1b	Naturalised
*Opuntia* sp.	1	Cactaceae	Shrub	NL	Naturalised
*Oxalis stricta* L.	1	Oxalidaceae	Herb	NL	Naturalised
*Phoenix reclinata* Jacq.	2	Arecaceae	Shrub	Native-alien	Casual
*Phyllostachys aurea* (AndrÃ©) RiviÃ¨re & C.RiviÃ¨re *	2	Poaceae	Grass	NL	Naturalised
*Physalis peruviana* L.*	9	Solanaceae	Herb	NL	Naturalised
*Physalis viscosa* L. *	29	Solanaceae	Herb	NL	Transformer
*Phytolacca dioica* L.	2	Phytolaccaceae	Woody	Category 3	Casual
*Pinus* sp.	1	Pinaceae	Woody	Category 2	Casual
*Plantago lanceolata* L. *	16	Plantaginaceae	Herb	NL	Invasive
*Populus alba* L.	1	Salicaceae	Woody	Category 2	Invasive
*Portulacaria afra* Jacq.	1	Portulacaceae	Succulent	Native-alien	Naturalised
*Prunus persica* (L.) Batsch	1	Rosaceae	Woody	NL	Casual
*Prunus* sp.	1	Salicaceae	Woody	NL	Casual
*Pteris vittata* L. *	3	Pteridaceae	Herb	NL	Casual
*Pueraria montana* (Lour.) Merr *	2	Fabaceae	Climber	Category 1a	Invasive
*Punica granatum* L.	3	Lythraceae	Shrub	NL	Naturalised
*Rapistrum rugosum* (L.) All.	2	Brassicaceae	Herb	NL	Naturalised
*Ricinus communis* L.*	3	Euphorbiaceae	Herb	Category 2	Naturalised
*Rivina humilis* L.*	24	Petiveriaceae	Herb	Category 1b	Transformer
*Robinia pseudoacacia* L.	4	Fabaceae	Woody	Category 1b	Naturalised
*Rumex* sp.	1	rumex sp	Shrub	NL	Naturalised
*Rumex usambarensis* (Dammer) Dammer *	9	Polygonaceae	Shrub	Category 1b	Naturalised
*Salix babylonica* L.	2	Polygonaceae	Woody	Category 2	Casual
*Senecio angulatus* L.f.	2	Asteraceae	Climber	Native-alien	Naturalised
*Schefflera arboricola* (Hayata) Merr.	1	Araliaceae	Shrub	Category 3	Naturalised
*Schefflera arboricola* (Hayata) Merr.	1	Araliaceae	Shrub	Category 3	Naturalised
*Schinus molle* L.	5	Anacardiaceae	Woody	NL	Naturalised
*Solanum chenopodioides* Lam.	3	Solanaceae	Herb	NL	Naturalised
*Solanum elaeagnifolium* Cav.*	24	Solanaceae	Shrub	Category 1b	Transformer
*Solanum mauritianum* Scop *	46	Solanaceae	Woody	Category 1b	Invasive
*Solanum nigrum* L.	4	Solanaceae	Herb	NL	Naturalised
*Styphnolobium japonicum* (L.) Schott	3	Fabaceae	Woody	NL	Casual
*Tagetes minuta* L.*	13	Asteraceae	Herb	NL	Naturalised
*Talinum paniculatum* (Jacq.) Gaertn.	1	Talinaceae	Herb	NL	Naturalised
*Tecoma stans* (L.) Juss. ex Kunth *	48	Bignoniaceae	Woody	Category 1b	Transformer
*Thaumatophyllum bipinnatifidum* (Schott ex Endl.) Sakur., Calazans & Mayo	1	Araceae	Shrub	NL	Casual
*Tephrosia grandiflora* (L’Her.) Pers	2	Fabaceae	Shrub	Native-alien	Casual
*Tipuana tipu* (Benth.) Kuntze *	17	Fabaceae	Woody	Category 3	Naturalised
*Tithonia rotundifolia* (Mill.) S.F.Blake *	3	Asteraceae	Herb	Category 1b	Naturalised
*Tithonia tubaeformis* (Jacq.) Cass *	9	Asteraceae	Herb	Category 1b	Invasive
*Ulmus parvifolia* Jacq.	1	Ulmaceae	Woody	NL	Naturalised
*Ulmus pumila* L.*	2	Ulmaceae	Woody	NL	Invasive
*Verbena bipinnatifida* Schauer *	34	Verbenaceae	Herb	NL	Invasive
*Verbena bonariensis* L.*	30	Verbenaceae	Herb	Category 1b	Naturalised
*Verbena brasiliensis* Vell.	8	Verbenaceae	Herb	Category 1b	Invasive
*Verbena rigida* Spreng *	10	Verbenaceae	Herb	Category 1b	Naturalised
*Yucca gloriosa* L.	1	Asparagaceae	Shrub	NL	Naturalised
