# Supplementary material for: Assessing Alien Plant Invasions in Urban Environments: A Case Study of Tshwane University of Technology and Implications for Biodiversity Conservation
Source: Plants (Basel). 2024 Mar 18;13(6):872. doi: 10.3390/plants13060872 (PMC10975853; doi:10.3390/plants13060872)
Supplement: Supplementary file 1 [file plants-13-00872-s001.zip › plants-2904916-supplementary.pdf]

Supplementary Materials 1. Output of Chi-square tests, comparing the number of species obtained from TUT across a) invasion status category, b) life forms, c) continent of origin d) reason of introduction and e) categories from the NEM:BA A&IS Regulations.

**Chi-square and Fisher's test results**		**Results**		
a) Comparing the number of species between invasion status	Dominant variable	Df	χ2	P
Naturalised vs invasive	Naturalized	1	014.79	<0.001
Naturalised vs transformer	Naturalized	1	17.02	<0.001
Naturalised vs casual	Naturalized	1	32.55	<0.001
b) Comparing the number of species between life forms				
Herbs vs woody	Herb	1	4.28	0.038
Herbs vs shrubs	Herb	1	6.72	0.009
Herbs vs climber	Herb	1	25.65	<0.001
Herbs vs grass	Herb	1	27.74	<0.001
c) Comparing the number of species between Continent of origin				
Asia vs South America	No difference	1	0.05	0.825
Asia vs North America	No difference	1	1.50	0.221
Asia vs Africa	Asia	1	9.54	0.002
Asia vs Central America	Asia	1	14.42	<0.001
Asia vs Europe	Asia	1	16.43	<0.001
Asia vs Australia	Asia	1	16.43	<0.001
d) Comparing the number of species between uses				
Horticulture vs medicinal	Horticulture	1	7.009	0.008
Horticulture vs edible	Horticulture	1	9.519	0.002
Horticulture vs weed	Horticulture	1	15.09	<0.001
Horticulture vs forestry	Horticulture	1	23.00	<0.001
Horticulture vs agriculture	Horticulture	1	31.64	<0.001
e) Comparing the number of species between NEM: BA Regulations categories				
Not listed vs category 1b	Not listed	1	3.747	<0.005
Not listed vs category 3	Not listed	1	21.53	<0.001
Not listed vs category 2	Not listed	1	30.53	<0.001
Not listed vs category 1a	Not listed	1	37.38	<0.001
